# Thrombocytopenia Impairs Host Defense Against *Burkholderia pseudomallei* (Melioidosis)

**DOI:** 10.1093/infdis/jiy541

**Published:** 2018-10-11

**Authors:** Emma Birnie, Theodora A M Claushuis, Gavin C K W Koh, Direk Limmathurotsakul, Nicholas P J Day, Joris J T H Roelofs, Jerry Ware, Baidong Hou, Alex F de Vos, Tom van der Poll, Cornelis van ‘t Veer, W Joost Wiersinga

**Affiliations:** 1 Center for Experimental and Molecular Medicine, Amsterdam University Medical Center (UMC), University of Amsterdam, The Netherlands; 2 Department of Medicine, University of Cambridge, United Kingdom; 3 Mahidol-Oxford Tropical Medicine Research Unit, Bangkok, Thailand; 4 Department of Tropical Hygiene, Faculty of Tropical Medicine, Mahidol University, Bangkok, Thailand; 5 Center for Tropical Medicine and Global Health, University of Oxford, United Kingdom; 6 Department of Pathology, Amsterdam UMC, University of Amsterdam, The Netherlands; 7 University of Arkansas for Medical Sciences, Little Rock; 8 Key Laboratory of Infection and Immunity, Institute of Biophysics, Beijing, China; 9 Division of Infectious Diseases, Academic Medical Center, Amsterdam UMC, University of Amsterdam, The Netherlands

**Keywords:** *Burkholderia pseudomallei*, melioidosis, pathogenesis, thrombocytopenia, platelets, sepsis

## Abstract

**Background:**

Infection with the gram-negative bacillus *Burkholderia pseudomallei* (melioidosis) is an important cause of pneumosepsis in Southeast Asia and has a mortality of up to 40%. We aimed to assess the role of platelets in the host response against *B. pseudomallei* infection.

**Methods:**

Association between platelet counts and mortality was determined in 1160 patients with culture-proven melioidosis. Mice treated with (low- or high-dose) platelet-depleting antibody were inoculated intranasally with *B. pseudomallei* and killed. Additional studies using functional glycoprotein Ibα–deficient mice were conducted.

**Results:**

Thrombocytopenia was present in 31% of patients at admission and predicted mortality in melioidosis patients even after adjustment for confounders. In our murine-melioidosis model, platelet counts decreased, and mice treated with a platelet-depleting antibody showed enhanced mortality and higher bacterial loads compared to mice with normal platelet counts. Low platelet counts had a modest impact on early-pulmonary neutrophil influx. Reminiscent of their role in hemostasis, platelet depletion impaired vascular integrity, resulting in early lung bleeding. Glycoprotein Ibα–deficient mice had reduced platelet counts during *B. pseudomallei* infection together with an impaired local host defense in the lung.

**Conclusions:**

Thrombocytopenia predicts mortality in melioidosis patients and, during experimental melioidosis, platelets play a protective role in both innate immunity and vascular integrity.

*Burkholderia pseudomallei* is a gram-negative environmental bacterium and the etiological agent of melioidosis, a life-threatening infection that often presents with pneumonia and sepsis and mainly occurs in Southeast Asia and northern Australia [1, 2]. Unpublished work shows us that about 5% of sepsis patients (using Sepsis-3 criteria) are diagnosed with culture-confirmed melioidosis in northeast Thailand. Despite appropriate antibiotic treatment, mortality rates remain high, ranging from 10% to 40% [1, 3]. Melioidosis has been proposed as a good model to study gram-negative sepsis [4, 5]. A 2016 epidemiological study estimated that there are 165000 cases and 89000 deaths annually, which suggests that the global burden of melioidosis is much larger than previously thought [6]. Additionally, due to its high lethality, severity of disease, intrinsic resistance to common antibiotics, and potential for easy dissemination, *B. pseudomallei* is declared as a Tier 1 biological threat agent [7]. New insights into the pathogenesis of melioidosis are urgently needed to develop novel adjunctive treatment strategies.

Platelets (anuclear cells derived from megakaryocytes) are of vital importance for hemostasis [8]. It has become clear that platelets also play an important role in inflammation and immunity [8–10]. Platelets have been described to express several immune-related receptors such as Toll-like receptors (TLRs), which are of importance for microbial surveillance and regulation of inflammatory and immune responses [11–13]. Indeed, platelets can aid in the host defense against infection [9, 11, 14] and can influence inflammation both in the lung and the systemic compartment [9, 15, 16]. In murine studies, platelets have been shown to alter immune responses by influencing leukocyte functions and recruitment [9, 17, 18]. In patients with sepsis, low platelet counts may dysregulate immune responses by decreasing leukocyte adhesion signaling [19].

We recently demonstrated that thrombocytopenia is a key feature of melioidosis and is correlated with mortality [20]. Von Willebrand factor (which can bind and activate platelets via platelet glycoprotein Ib [GPIb]) levels are elevated in patients with melioidosis [20]. We therefore sought to further define the role of platelets in melioidosis, using observational data from a large cohort of melioidosis patients and from clinically relevant murine models of melioidosis.

## METHODS

### Cohort Study

Patients presenting to Sappasithiprasong Hospital, Ubon Ratchathani, northeast Thailand, with culture-confirmed melioidosis were prospectively included between 1 January 2002 and 31 December 2006. This cohort has been previously described elsewhere [21]. Patients were stratified into 3 groups according to platelet counts at presentation; low platelet count (<100 × 10^9^/L), intermediate-low platelet counts (100–149 × 10^9^/L), or normal platelet counts (≥150 × 10^9^/L). Boundaries were based on previous studies [19, 22]. The primary study outcome was in-hospital mortality, but we also predefined 3 secondary outcomes: hypotension, acute kidney injury, and respiratory failure (also see Supplementary Materials).

### Animals

For the platelet depletion experiment, specific pathogen-free C57Bl/6 mice (Charles River, France) were used. Platelet-specific MyD88 knock-out (Plt-MyD88^–/–^) mice were generated as previously described [23]. Human IL4R/GPIbα mice are knock-out for mouse GPIbα, without the associated macrothrombocytopenia [24] (also see Supplementary Materials).

### Ethics Statement

Approval was obtained from the Ethical and Scientific Review subcommittee of the Thai Ministry of Public Health to use information collected during the cohort study. Written informed consent was obtained from all subjects by a native Thai speaker. Parents/guardians also provided written informed consent on behalf of child participants. All procedures performed were in accordance with the Helsinki Declaration of 1975 (revised 1983). The Institutional Animal Care and Use Committee of the Academic Medical Center approved all experiments (DIX 21) and ethical approval was obtained to use *B. pseudomallei* strain 1026b for animal experiments (08-150). Experiments were carried out in accordance with the Dutch Experiments on Animals Act.

### Experimental Study Design

Melioidosis was induced by intranasal inoculation with *B. pseudomallei* 350–500 colony-forming units in 50 μL isotonic saline, as previously described [25–27]. Two hours before infection, mice were intravenously injected with platelet-depleting antibody (polyclonal antimouse GPIbα, 0.4 or 2 µg/g) or control immunoglobulin G (both Emfret Analytics, Eibelstadt, Germany) [14]. To assess the effect of platelet depletion on survival, mice were observed for 10 days (n = 20 per group) and clinical symptoms were scored with an independent animal biotechnician, unaware of group allocation, as previously described [14] (also see Supplementary Materials).

Flow cytometry, pathology [14, 28], and protein measurements are shown in the Supplementary Materials.

### Statistical Analysis

In the human cohort, analyses were performed using Stata/SE version 9 software (StataCorp). Differences between the 3 patient groups were compared using Fisher exact test for categorical variables. Difference in age was analyzed by analysis of variance; days to infective symptoms prior to presentation by Kruskal–Wallis test; sex by χ^2^ test; and risk factors, organ involvement, and distribution of disease by Fisher exact test. Patient outcomes were calculated using χ^2^ test. Time to death to 28 days was analyzed using the Kaplan–Meier method. Logistic regression models were used to adjust for confounders identified using a conceptual hierarchical framework (Supplementary Figure 1) [29]. Parameters were chosen on the basis of whether they were possible confounders for the effect of thrombocytopenia on mortality. For murine studies, see the Supplementary Materials.

## RESULTS

### Clinical Melioidosis Is Associated With Thrombocytopenia

We analyzed 1160 patients with a first hospital admission for culture-positive melioidosis. All patients were prospectively identified, were aged ≥15 years, and presented to Sappasithiprasong Hospital, Thailand, as described previously [21]. The lungs were the most frequent organs infected (in approximately 40% of patients). Patients were on average 51 years old (range, 18–86 years) and 61% were male. Three hundred sixty-two patients (31%) showed thrombocytopenia, that is, platelets counts <150 × 10^9^/L. There were 199 patients (17%) with low platelet counts of <100 × 10^9^/L, 163 patients (14%) with intermediate-low platelet counts between 100 and 149 × 10^9^/L, and 798 (69%) with normal platelet counts (≥150 × 10^9^/L). Baseline characteristics between groups were largely similar; in the groups with low and intermediate-low platelet counts, patients presented more with bacteremia and multiorgan disease (*P* < .001), and the duration of symptoms prior to presentation was shorter than in the group with a normal platelet count (*P* < .001; Table 1).

**Table 1.** Patient Demographics and Outcome of 1160 Patients With a First Hospital Admission for Culture-Positive Melioidosis

Characteristic	Low Platelets(<100 × 10^9^/L)	Intermediate-Low Platelets (100–149 × 10^9^/L)	Normal Platelets (≥150 × 10^9^/L)	*P* Value
Patients, No.	199	163	798	
Baseline characteristics				
Age, y, mean (SD)	51 (14)	53 (14)	50 (14)	.20
Male sex	126 (63)	99 (61)	437 (55)	.06
Days of infective symptoms prior to presentation, median (IQR)	7 (5–7)	7 (5–7)	10 (10−14)	<.001
Risk factors for melioidosis				
Rice farmer	159 (80)	130 (80)	616 (77)	.64
Known diabetes	62 (31)	46 (28)	302 (38)	.04
Chronic kidney disease	24 (12)	24 (15)	51 (6)	<.001
Nephrolithiasis	8 (4)	9 (6)	39 (5)	.78
Corticosteroid use	11 (6)	6 (4)	32 (4)	.57
Thalassemia	3 (2)	2 (1)	12 (2)	>.999
Malignancy	0 (0)	1 (1)	2 (<1)	.43
Chronic liver disease	3 (2)	3 (2)	6 (1)	.26
Organ involvement				
Pneumonia	80 (40)	70 (43)	311 (39)	.63
Skin and soft tissue	18 (9)	22 (14)	182 (23)	<.001
Urinary tract	33 (17)	23 (14)	73 (9)	.007
Liver abscess	8 (4)	11 (7)	89 (11)	.003
Spleen abscess	19 (10)	14 (9)	92 (12)	.50
Septic arthritis	10 (5)	9 (6)	69 (9)	.14
Distribution of disease				
Bacteremia	159 (80)	120 (74)	397 (50)	<.001
Single organ disease	92 (46)	84 (52)	501 (63)	<.001
Outcome				
Hypotension	126 (63)	81 (50)	214 (27)	<.001
Respiratory failure	118 (59)	83 (51)	196 (25)	<.001
Acute kidney injury	104 (52)	67 (41)	182 (23)	<.001
Died in hospital	155 (78)	98 (60)	246 (31)	<.001

Data are presented as No. (%) unless otherwise indicated. Difference in age was analyzed by analysis of variance; days to infective symptoms prior to presentation by Kruskal–Wallis test; sex by χ^2^ test; and risk factors, organ involvement, and distribution of disease by Fisher exact test. Patient outcomes were calculated using χ^2^ test.

Abbreviations: IQR, interquartile range; SD, standard deviation.

### Thrombocytopenia Predicts Mortality in Melioidosis Patients

**Figure 1.**
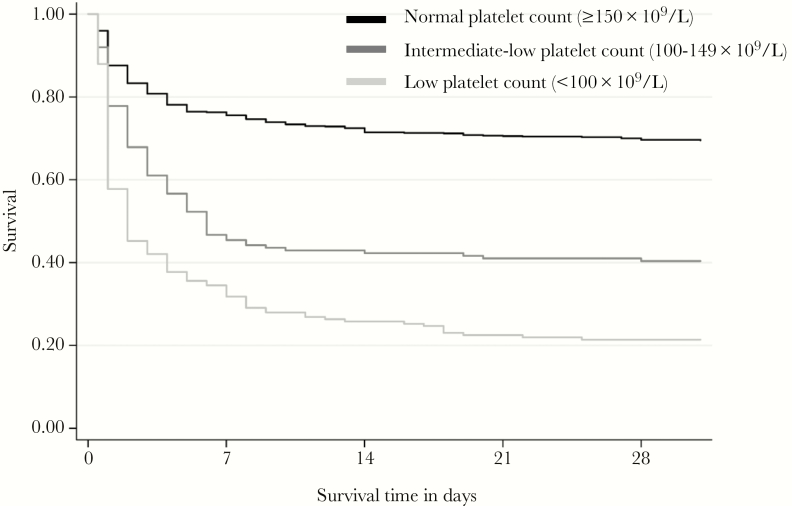
Thrombocytopenia is associated with increased mortality in melioidosis patients. Kaplan–Meier survival curves of 1160 patients with melioidosis stratified according to platelet counts on admission. Patient were stratified in groups with low platelet count (<100 × 10^9^/L) in light gray, intermediate-low platelet count (100–149 × 10^9^/L) in gray, or normal platelet count in black (≥150 × 10^9^/L). *P* < .001 for comparison between 3 groups for survival analysis.

Thrombocytopenic patients developed more respiratory failure, hypotension, and acute kidney injury during admission (*P* < .001 vs patients with normal platelet counts; Table 1). Overall mortality was 43%, and low (<100 × 10^9^/L) and intermediate-low (100–149 × 10^9^/L) platelet counts on admission were associated with higher in-hospital mortality (*P* < .001; Table 1). In-hospital mortality was highest in the low platelet count group (78%), followed by the intermediate-low group (60%) and normal platelet count group (31%). These findings were reproduced in the survival analysis up to 28 days postadmission (*P* < .001; Figure 1). Low platelet counts <100 × 10^9^/L (odds ratio [OR], 7.90; 95% confidence interval [CI], 5.5–11.4) and intermediate-low platelet counts 100–149 × 10^9^/L (OR, 3.38; 95% CI, 2.4–4.8) were associated with increased mortality compared to patients with normal platelet counts (≥150 × 10^9^/L). This association persisted after correcting for these confounders, with increased adjusted ORs (aORs) in patients with low platelet counts (aOR, 7.98; 95% CI, 5.5–11.6) and intermediate-low platelet counts (aOR, 3.40; 95% CI, 2.4–4.8) compared to normal platelet counts. Important confounders were selected by hierarchical pathway analyses: sex, age, rice farming (malnutrition), liver cirrhosis, malignancy, and diabetes mellitus (Supplementary Figure 1). These results show that thrombocytopenia is associated with poor outcome and predicts mortality in melioidosis patients, even after correcting for important confounders. Disease severity scores of our patients were, however, not collected, hampering our ability to correct for this important confounder.

### Platelet Depletion Impairs Survival and Host Defense in Experimental Melioidosis

**Figure 2.**
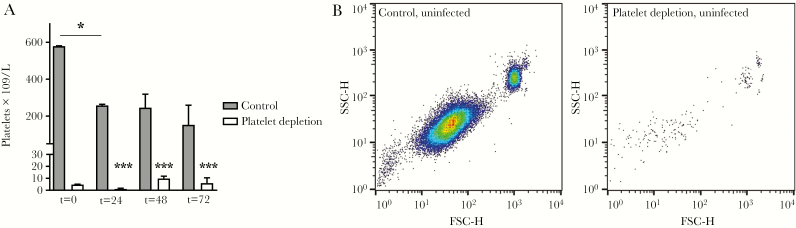
Experimental melioidosis is associated with thrombocytopenia and effect of anti-GPIbα on platelet counts. Mice were treated with anti-GPIbα (platelet depletion) or immunoglobulin G (IgG) control (both 0.4 µg/g) and infected with *Burkholderia pseudomallei* via the airway and killed after 24, 48, or 72 hours or killed uninfected (t = 0 hours). *A*, Platelet counts before (t = 0 hours) and after (t = 24, 48, or 72 hours) infection. *B*, Representative log-scale scatterplots of CD61-positive platelets in blood of uninfected control and anti–GPIbα-treated mice. Data are represented as bars (median with interquartile range). n = 8 mice per group. *P < .05, ***P < .001 vs IgG control or vs uninfected mice. Abbreviations: FSC-H, forward scatter height; SSC-H, side scatter height.

**Figure 3.**
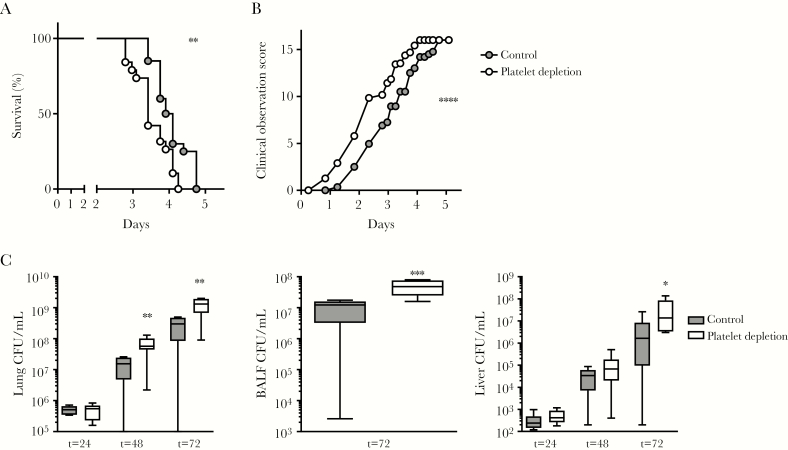
Thrombocytopenia impairs survival and enhances bacterial growth during *Burkholderia pseudomallei* pneumonia-derived sepsis. Mice were treated with anti-GPIbα (platelet depletion) or immunoglobulin G (IgG) control (both 0.4 µg/g) and infected with *B. pseudomallei* via the airway and killed after 24, 48, or 72 hours or were observed in a survival experiment. Survival (*A*) and clinical observation score (*B*). *C*, Bacterial quantification of indicated organs. Data are expressed as box-and-whisker plots depicting the smallest observation, lower quartile, median, upper quartile, and largest observation. n = 20 per group for survival experiment and n = 8 mice per group for bacterial quantification. *P < .05, **P < .01, ***P < .001 vs IgG control. Abbreviations: BALF, bronchoalveolar lavage; CFU, colony-forming units.

As human studies are limited in their ability to investigate causality, we conducted murine studies to further assess the contribution of platelets to the host response. We used a clinically relevant model, which starts with a low dose of *B. pseudomallei* given intranasally, with gradually increasing bacterial counts in the lung and dissemination to distant organs. Similar to patients with melioidosis, mice infected with *B. pseudomallei* showed a decline in platelet counts (median platelet counts at 72 hours after infection, 152 × 10^9^/L; interquartile range [IQR], 112–260 × 10^9^/L); noninfected mice had median platelet counts of 579 × 10^9^/L (IQR, 529–581 × 10^9^/L) (*P* < .05 for infected vs uninfected; Figure 2A). To investigate the role of platelets during melioidosis, mice were depleted of platelets toward levels of <5% of normal using anti-GPIbα antibody as we have described before [14, 30]. Mice remained thrombocytopenic during infection (Figure 2A and 2B). During murine melioidosis, platelet-depleted mice showed increased mortality and an increased clinical observation score, a readout for disease severity (*P* < .001 vs controls; Figure 3A and 3B). To investigate if differences in outcome were mediated by changes in host defense, *B. pseudomallei* burden was assessed at multiple time points after infection. Platelet depletion increased bacterial numbers in lung, bronchoalveolar lavage fluid (BALF), and liver (*P* < .05, *P* < .01, and *P* < .001, respectively, vs controls; Figure 3A and 3B), but not blood (Supplementary Figure 2). These data indicate that during murine melioidosis, platelets are important for outcome and host defense.

### Platelets Do Not Exert Direct Antibacterial Effect on *B. pseudomallei* Growth

To assess if thrombocytopenia could directly influence bacterial growth, whole blood of platelet-depleted and control mice was incubated with *B. pseudomallei* and ex vivo bacterial growth assessed. Blood of both groups showed similar bacterial growth (Supplementary Figure 3*A*). Likewise, no differences were found in *B. pseudomallei* growth rate between human platelet-rich and platelet-poor plasma (Supplementary Figure 3*B*). Together, these data suggest that platelets do not directly influence *B. pseudomallei* growth.

### Modest Impact of Platelets on Early Pulmonary Neutrophil Influx

**Figure 4.**
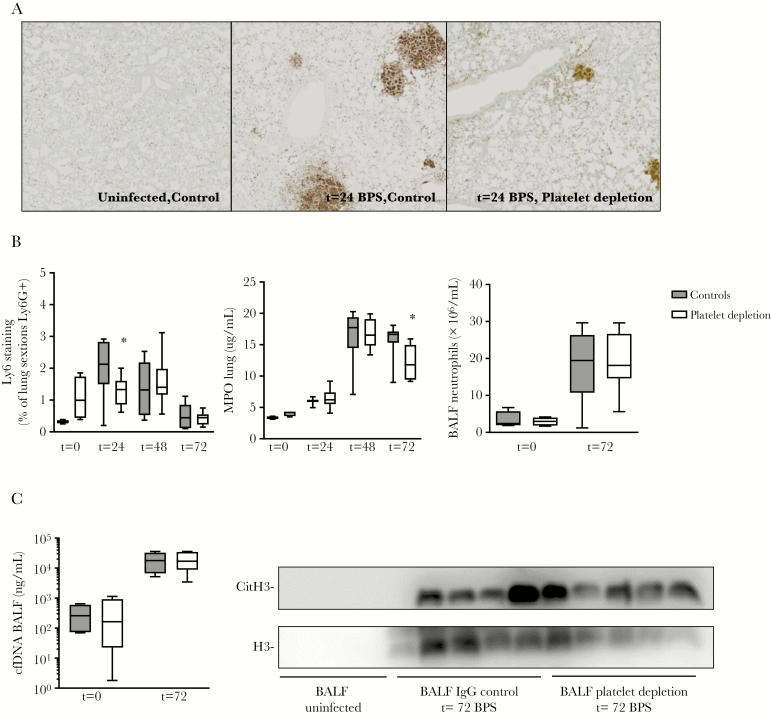
Platelet depletion affects early neutrophil recruitment but not neutrophil extracellular trap formation. Mice were treated with anti-GPIbα (platelet depletion) or immunoglobulin G (IgG) control (both 0.4 µg/g) and infected with *Burkholderia pseudomallei* via the airway and killed after 24, 48, or 72 hours or killed uninfected. *A*, Representative images of Ly6G staining lung sections. *B*, Neutrophil influx; measured by Ly6G staining quantification, myeloperoxidase levels in lung, and neutrophil counts in bronchoalveolar lavage fluid (BALF). *C*, Cell-free DNA and citrullinated histone 3 levels in BALF. Data are expressed as box-and-whisker plots depicting the smallest observation, lower quartile, median, upper quartile, and largest observation. n = 8 mice per group. *P < .05 vs IgG control. Abbreviations: BALF, bronchoalveolar lavage fluid; BPS, *B. pseudomallei*; cfDNA, cell-free DNA; CitH3, citrullinated histone 3; IgG, immunoglobulin G; MPO, myeloperoxidase.

As neutrophils influence antibacterial defense during melioidosis [31, 32], we assessed if platelets mediate their protective effects via neutrophil recruitment [33]. By quantification of Ly-6G–positive cells in lung tissue (Figure 4A and 4B) and by measuring myeloperoxidase (MPO) (Figure 4B) in whole lung homogenates, we determined lung neutrophil influx for both platelet-depleted and control mice at set time-points postinfection. Platelet depletion had a modest impact on early neutrophil recruitment, as reflected by reduced lung neutrophil Ly6G staining 24 hours after infection (*P* < .05 vs controls; Figure 4A and 4B). MPO levels were reduced in thrombocytopenic mice at 72 hours postinfection (*P* < .05 vs controls; Figure 4B); however, neutrophil counts in BALF and lung Ly6G staining were no different between groups at this late time point (Figure 4B and Supplementary Figure 4). Platelets are potent inducers of neutrophil extracellular traps (NETs), which are used by neutrophils to ensnare and kill *B. pseudomallei* [34, 35]. To assess the role of platelets in NET formation during melioidosis in vivo, we determined cell-free DNA (cfDNA) and citrullinated histone 3 levels in BALF (Figure 4C). *Burkholderia pseudomallei* was shown to be a potent inducer of NET formation, which was, however, similar between groups (Figure 4C). We therefore conclude that platelet depletion has a modest impact on early neutrophil recruitment, but not on NET formation during murine melioidosis.

### Platelet Depletion Increases Local and Systemic Inflammatory Responses

Platelets can influence inflammatory responses of other cells, for example, monocytes [33]. To investigate if this also mediated the observed protective effects of platelets during melioidosis, we assessed local and systemic cytokine and chemokine production. Platelet depletion increased cytokine and chemokine levels in both lung and plasma (most notably tumor necrosis factor α and CXCL-2; Supplementary Table 1), in part likely driven by higher bacterial loads. This aggravated cytokine response in platelet-depleted mice was not reflected in altered systemic organ damage, which is a hallmark feature of sepsis. There were no differences in liver damage (as scored by a blinded pathologist) or plasma aspartate aminotransferase and alanine aminotransferase levels between groups (Supplementary Figure 5*A–D*).

### Platelet Depletion Toward a Level of <1% of Normal Also Impairs Host Defense During Melioidosis

We have recently shown that during *Klebsiella pneumoniae*–induced pneumosepsis, platelet depletion toward a level of <1% of normal has a more pronounced phenotype compared with platelet depletion toward a level of <5% of normal [14]. To investigate if platelet counts <1% would show similar effects on host defense, inflammatory responses, and vascular integrity, we treated mice with a high-dose (2 µg/g) platelet-depleting antibody and assessed these parameters during melioidosis. Platelet counts <1% also increased bacterial loads in lung and liver (Supplementary Figure 6*A*). Additional lung MPO levels were decreased; however, Ly6G staining was similar between groups (Supplementary Figure 6*B*). Despite an increased local and systemic inflammatory response (Supplementary Table 2), distant organ damage was similar (Supplementary Figure 6*B* and 6*C*). These data indicate that both platelet depletion <1% and <5% impair host defense during melioidosis.

### Platelet Depletion Impairs Vascular Integrity During Experimental Melioidosis

**Figure 5.**
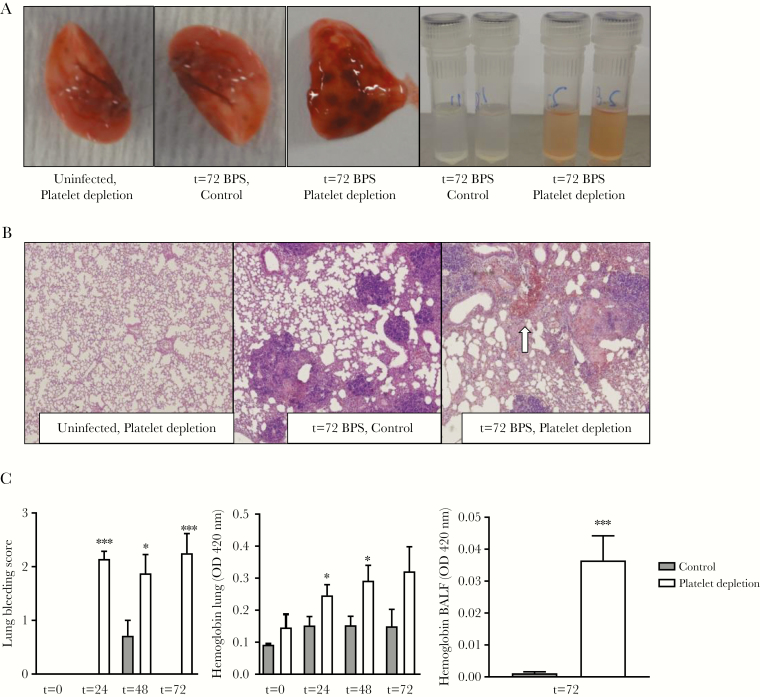
Thrombocytopenia results in lung bleeding at the site of infection. Mice were treated with anti-GPIbα (platelet depletion) or immunoglobulin G (IgG) control (both 0.4 µg/g) and infected with *Burkholderia pseudomallei* via the airway and killed after 24, 48, or 72 hours or killed uninfected. *A*, Representative photographs of naive or infected lungs and bronchoalveolar lavage fluid (BALF). *B*, Representative microphotographs of hematoxylin and eosin (H&E)–stained tissue sections (original magnification ×40), bleeding indicated by arrow. *C*, Quantification of lung bleeding; both scored on H&E-stained tissue sections by a pathologist blinded for groups and hemoglobin measurement in 50-fold diluted lung homogenates or BALF. Data are represented as bars (mean with standard error of the mean). n = 8 mice per group. *P < .05, ***P < .001 vs IgG control. Abbreviations: BALF, bronchoalveolar lavage fluid; BPS, *B. pseudomallei*; OD, optical density.

Platelets are of vital importance for hemostasis and are known to prevent bleeding during pneumosepsis [14]. In line with this, we found that—where uninfected platelet depleted mice showed no signs of bleeding—platelet depletion (<5%) induced lung bleeding during melioidosis (Figure 5A). Bleeding started at 24 hours after infection as measured by increased lung and BALF hemoglobin levels and lung pathology bleeding scores (*P* < .05 vs control; Figure 5A–C).

Furthermore, we investigated if impaired coagulation might explain the effects of platelet depletion on host defense or vascular integrity, as platelet phosphatidylserine exposure aids in the conversion of coagulation factors [9]. A decrease in platelet counts may also indicate pathologic coagulation activation [10], which can contribute to complications in melioidosis such as disseminated intravascular coagulation and multiple organ failure [36, 37]. Mice infected with *B. pseudomallei* demonstrated strong activation of the coagulation system, as reflected by high plasma levels of thrombin–antithrombin complexes (TATc) and elevated levels of D-dimer (Supplementary Figure 7*A–D*). This is in line with findings in patients with melioidosis [38]. Platelet depletion (<5%) further increased lung D-dimer and TATc levels in lung and plasma (Supplementary Figure 7*A–D*) when compared to controls. The increased accumulation of fibrin products in the lung in the thrombocytopenic mice can be due to extravascular formation of fibrin as a result of the bleeding or may be due to increased coagulation as a results of the increased bacterial burden and inflammatory response (Figure 3 and Supplementary Table 1). Platelet depletion <1% also induced lung bleeding during melioidosis (Supplementary Figure 6*C*). Taken together, these results show that platelet depletion impairs vascular integrity in the lung during melioidosis.

### Mice Lacking Platelet GPIbα, but Not Platelet TLR Signaling, Display Impaired Local Host Defense During Melioidosis

**Figure 6.**
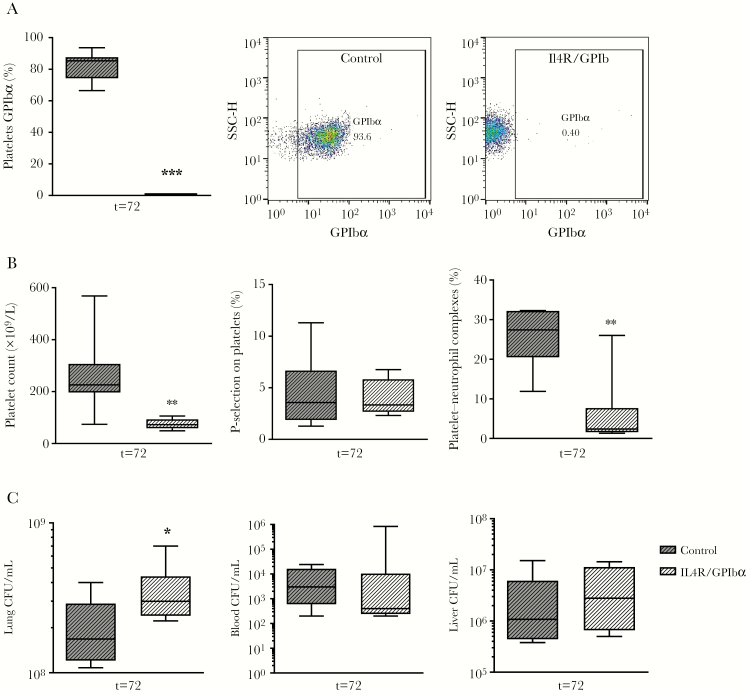
Platelet GPIb deficiency decreases platelet counts and leads to increased bacterial growth in the lung during experimental melioidosis. IL4R/GPIbα or control mice were infected with *Burkholderia pseudomallei* via the airway and killed after 72 hours. *A*, GPIb expression on platelets. *B*, Platelet counts, P-selectin expression, and platelet–neutrophil complex formation. *C*, Bacterial quantification in indicated organs. Data are expressed as box-and-whisker plots depicting the smallest observation, lower quartile, median, upper quartile, and largest observation. n = 8 mice per group. *P < .05, **P < .01, ***P < .001 vs IgG control. Abbreviations: CFU, colony-forming units; SSC-H, side scatter height.

To assess which platelet receptors were involved in the protective effect of platelets during melioidosis, we investigated mice lacking platelet TLR signaling or GPIbα. Platelet TLRs and GPIbα can influence platelet–leukocyte interactions and cytokine release [11, 15, 39]. Moreover, it was recently shown that platelet GPIbα can influence host defense during *K. pneumoniae*–induced pneumosepsis [17]. To investigate platelet-specific TLR signaling, we used Plt-MyD88^–/–^ mice, which lack the crucial TLR signaling protein MyD88 only in platelets and megakaryocytes [23]. Total MyD88^–/–^ mice have impaired host defense during *B. pseudomallei* infection [40]. However, infected Plt-MyD88^–/–^ and littermates had similar bacterial loads in all organs during melioidosis (Supplementary Figure 8*A*). Moreover, platelet activation, platelet–leukocyte interactions and thrombocytopenia were similar between both groups (Supplementary Figure 8*B*). To investigate the role of platelet GPIbα, IL4R/GPIbα mice (that lack GPIbα, but without the associated macrothrombocytopenia) [24] were infected with *B. pseudomallei*. IL4R/GPIbα mice had no GPIbα expression (*P* < .001 vs controls; Figure 6A) and reduced platelet counts during infection (*P* < .01 vs controls; Figure 6B). Platelet activation as measured by P-selectin on platelets was similar between mice strains during infection (Figure 6B), platelet neutrophil-complexes were reduced in IL4R/GPIbα compared to controls (*P* < .01 vs controls; Figure 6B). IL4R/GPIbα mice showed increased lung bacterial loads compared to controls in our experimental melioidosis model. This was not to a similar extent as observed in the experiments in which platelets were depleted with anti-GPIbα antibody (*P* < .05 vs controls; Figure 6C) as bacterial loads in distant organs were unaffected (Figure 6C). Neither Plt-MyD88^–/–^ mice or IL4R/GPIbα mice showed increased lung bleeding during melioidosis (Supplementary Figure 9). These results show that GPIbα, but not platelet TLR signaling, contributes to the local host defense against *B. pseudomallei*.

## DISCUSSION

Here, we show that during clinical melioidosis, thrombocytopenia is an independent predictor of mortality, and that in murine melioidosis, platelet depletion reduces survival and impairs host defense. Our associations between thrombocytopenia and mortality in melioidosis patients are in line with our previous findings in a much smaller patient cohort [20] as well as other studies looking at sepsis [19, 41, 42]. The correlations between platelet counts and mortality might be a reflection of disease severity. This is underlined by the finding that melioidosis patients with low platelet counts developed more hypotension, respiratory failure, and kidney failure. However, thrombocytopenia is associated with altered immune responses independent of disease severity, as we have shown previously in another cohort of sepsis patients [19]. Disease severity scores of our patients were, however, not collected, hampering our ability to correct for this important confounder.

In contrast to observational cohort studies, murine studies can be used to investigate the direct contributing effect of platelets on melioidosis outcome. Similar to the human setting, murine melioidosis led to marked thrombocytopenia and platelet depletion toward levels <5% of normal was associated with increased mortality. Moreover, platelets directly contributed to host defense against *B. pseudomallei*, both at the local site of infection as well as distant organs, such as the liver. Of interest, platelet depletion increases local and systemic inflammatory responses during experimental melioidosis. These findings are in line with other murine studies of gram-negative sepsis using *Escherichia coli* [15, 16] and *K. pneumoniae* [14], as well as our previous findings in patients with sepsis [19].

Neutrophils recruited to the site of infection can influence outcome during melioidosis [31], mostly during late-stage infection. Additionally, interactions between platelets and neutrophils can influence bacterial killing [11]. During melioidosis, platelet depletion modestly impaired early neutrophil recruitment to the lung. These findings are in line with previous studies showing that platelets can influence recruitment of neutrophils to the site of infection during *Pseudomonas* infection and during a (polymicrobial) cecal ligation and puncture model [43, 44] However, during *Klebsiella* infection, platelets did not influence neutrophil recruitment [14]. Interestingly, we observed an effect of platelets on antibacterial defense 48 hours after infection, whereas previous studies showed an effect of neutrophils 72 hours after *B. pseudomallei* infection [31]. We made use of a platelet depletion antibody and depleted platelets to <5% of normal. Although this mice model is used often to examine the effect of platelets on the host defense, it is important to note that the thrombocytopenia in those mice is more severe compared with the much more modest levels of thrombocytopenia in patients with melioidosis. Previous studies have shown that platelets can induce NET formation and this can influence bacterial growth of *E. coli* and *Staphylococcus aureus* [11, 45]. During melioidosis, however, platelet depletion did not impair NET formation, as assessed by cfDNA and citrullinated histone 3 levels. Earlier, we reported that compromised NETs (by DNase treatment) also did not affect outcome in murine melioidosis [34].

Previous studies have shown that platelet TLR signaling was important for restriction of *E. coli* growth [11], but during infection with *Streptococcus pneumoniae*, *K. pneumoniae*, and *B. pseudomallei* [23, 46], platelet TLR signaling did not contribute to host defense. These differences might be explained by differences in bacteria, the intracellular nature of *B. pseudomallei*, or the model (acute high dose vs slowly growing).

Mice lacking platelet GPIbα did show impaired host defense in the lung. IL4R/GPIbα mice showed increased bacterial burden in the lung after 3 days of infection, but not to the extent of that in platelet-depleted mice. IL4R/GPIbα mice are GPIbα-deficient mice without the associated macrothrombocytopenia [24]; however, we found that during melioidosis, platelet counts were still reduced in IL4R/GPIbα mice compared with controls. The contribution of the lower platelet counts on the phenotype seen remains to be established. In line with a protective effect of platelet GPIbα, a recent study also found a protective role for platelet GPIbα during gram-negative pneumosepsis caused by *K. pneumoniae*, using a GPIbα blocking antibody [17].

Platelets protect against bleeding, specifically at the site of infection and inflammation [14, 47]. Platelet depletion resulted in bleeding in the lung during *B. pseudomallei* infection, a finding consistent with previous reports in *Klebsiella* and *Streptococcus* pneumosepsis [14, 48]. However, in contrast to *Klebsiella* infection [14], melioidosis also induced severe bleeding when platelet counts were <5%. Possibly, *B. pseudomallei* infection of cells causes more severe damage to tissue as well as vascular integrity, which renders more platelets needed to prevent bleeding. Also, in platelet-depleted mice lung bleeding was already seen at an early time point (24 hours after infection) in *B. pseudomallei* infection. It is possible that this early lung bleeding influences adequate host defense and thereby contributes to the differences in antibacterial response seen between control and platelet-depleted mice. In addition, increased bacterial growth and cytokine levels seen could be a direct consequence or cause the bleeding seen in the thrombocytopenic mice, as the release of heme and transferrin free iron can activate and deteriorate the immune system [49, 50]. In human melioidosis, bleeding complications are rarely observed [1–3]. The importance of the risk of bleeding in melioidosis patients remains to be elucidated.

In conclusion, we found that thrombocytopenia predicted mortality in melioidosis patients even after adjustment for confounders and that, in murine melioidosis, platelet depletion severely hampered survival, host defense, and vascular integrity.

## Supplementary Data

Supplementary materials are available at *The Journal of Infectious Diseases* online. Consisting of data provided by the authors to benefit the reader, the posted materials are not copyedited and are the sole responsibility of the authors, so questions or comments should be addressed to the corresponding author.

## Supplementary Material

Supplementary Figures and Tables

Supplementary Material and Methods

## Notes

***Acknowledgments.*** We thank Joost Daalhuisen, Marieke ten Brink, and Jasmin Ersoz for technical assistance during the animal experiments and Regina de Beer for preparations of lung tissue slides.

***Disclaimer.*** The funders had no role in study design, data collection and analysis, decision to publish, or preparation of the manuscript.

***Financial support.*** This work was supported by the Academic Medical Center PhD scholarship award (number 22162 to E. B.); the Royal Society of Tropical Medicine and Hygiene (award numbers GR000559 to E. B. and GR000697 to T. A. M. C.); and the Landsteiner Foundation for Blood Transfusion Research (grant number 1351 to T. A. M. C.). W. J. W is financially supported by the Netherlands Organization for Scientific Research (VIDI grant number 91716475) and the Marie Curie Skledowska Innovative Training Network (“European Sepsis Academy”).

***Potential conflicts of interest.*** All authors: No reported conflicts of interest. All authors have submitted the ICMJE Form for Disclosure of Potential Conflicts of Interest. Conflicts that the editors consider relevant to the content of the manuscript have been disclosed.

Presented in part: South-East Melioidosis Congress, Colombo, Sri Lanka, 2017; International Society on Thrombosis and Haemostasis, Berlin, Germany, 2017; European Shock Society, Paris, France, 2017; and European Melioidosis Congress, Oxford, United Kingdom, 2017.
