# Supplementary material for: Thrombocytopenia Impairs Host Defense Against Burkholderia pseudomallei (Melioidosis)
Source: J Infect Dis. 2018 Oct 11;219(4):648–59. doi: 10.1093/infdis/jiy541 (PMC6350952; doi:10.1093/infdis/jiy541)
Supplement: Supplementary Figures and Tables [file jiy541_supplemental_figures_and_tables.docx]

**Supplementary Figures to 'Thrombocytopenia impairs host defense against *Burkholderia pseudomallei* (melioidosis)'**

|  | **Lung homogenates** | | **Plasma** | |
| --- | --- | --- | --- | --- |
| ng/mL | Control | Platelet depletion | Control | Platelet depletion |
| **24 h** | | | | |
| TNF-α | 3.8 (0.1-8.5) | 3.3 (0.2-9.3) | 0.2 (0.1-0.2) | 0.1 (0.1-0.2) |
| IL-6 | 23.1 (6.6-36.9) | 41.0 (30.6-43.5) | 0.2 (0.2-0.2) | 0.2 (0.2-1.0) |
| IFN-γ | 1.2 (0.8-2.1) | 2.3 (1.0-3.0) | b.d. | b.d |
| CXCL2 | 20.3 (13.5-26.7) | 24.1 (14.1-31.4) | n.d. | n.d. |
| CCL2 | n.d. | n.d. | 3.1 (2.8-3.7) | 2.8 (2.1-3.4) |
| **48 h** | | | | |
| TNF-α | 20.6 (12.7-23.6) | 23.8 (23.8-31.1)***** | 0.6 (0.4-1.0) | 1.2 (1.0-1.4)***** |
| IL-6 | 82.0 (41.6-10.8) | 113.3 (91.4-135.0) | 2.4 (1.6-13.1) | 18.6 (10.3-32.7) |
| IFN-γ | 2.7 (2.5-2.9) | 2.5 (2.1-3.1) | 0.1 (0-0.2) | 0.3 (0.2-0.6)***** |
| CXCL2 | 166.2 (112.6-194.5) | 310.8 (215.6-343.1)****** | n.d. | n.d. |
| CCL2 | n.d. | n.d. | 5.3 (2.3-11.7) | 4.0 (1.5-12.3) |
| **72 h** | | | | |
| TNF-α | 23.2 (4.1-27.3) | 40.0 (27.7-41.4)****** | 2.1 (0.7-7.1) | 7.1 (4.7-10.9)***** |
| IL-6 | 77.3 (35.0-88.9) | 79.0 (7.6 -112.5) | 50.1 (6.9-157.1) | 145.1 (100.6-277.1) |
| IFN-γ | 2.3 (1.8-2.6) | 2.3 (2.1-2.8) | 0.09 (0-0.3) | 0.5 (0.2-2.0) |
| CXCL2 | 294.8 (200.5-465.6) | 737.4 (417.3-873.3)***** | n.d. | n.d. |
| CCL2 | n.d. | n.d. | 9.6 (2.9-16.2) | 12.2 (10.3-19.0) |

**S1 Table. Cytokine and chemokine levels in lung homogenate and plasma during experimental melioidosis.** Mice were treated with anti-GPIbα or IgG control (0.4µg/g), infected with *B. pseudomallei* via the airway and sacrificed after 24, 48 or 72 hours. Cytokine and chemokine levels in lung homogenate and plasma. Values are in ng/mL and presented as median (interquartile range). N=8 mice per group. **P* < .05, ***P* < .01 vs IgG control. Abbreviations: b.d.= below detection, n.d= not determined.

|  | **Lung homogenates (t=72)** | | **Plasma (t=72)** | |
| --- | --- | --- | --- | --- |
| ng/mL | Control | Platelet depletion (high dose) | Control | Platelet depletion (high dose) |
| TNF-α | 30.7 (21.2-34.1) | 64.2 (49.1-72.3)** | 1.9 (1.4-3.4) | 7.9 (7.1-9.6)** |
| IL-6 | 47.2 (35.3-51.0) | 96.9 (89.2-130.3)** | 22.0 (16.6-43.8) | 241.8 (178.5-299.6)* |
| IFN-γ | 1.7 (1.5-2.2) | 2.4 (2.0-3.0) | 0.1 (0.0-0.8) | 0.0 (0.0-1.2) |
| CXCL2 | 271.3 (154.5-357.7) | 658.8 (376.8-801.4)* | n.d. | n.d. |
| CCL2 | n.d. | n.d. | 3.3 (1.4-5.1) | 1.2 (0.9-1.2) |

**S2 Table. Cytokine and chemokine levels in lung homogenate and plasma during murine melioidosis in experiments in which the high dose of anti-GPIbα was used.** Mice were treated with anti-GPIbα or IgG control (2.0µg/g), infected with *B. pseudomallei* via the airway and sacrificed after 72 hours. Cytokine and chemokine levels in lung homogenate and plasma. Values are in ng/mL and presented as median (interquartile range). N=8 mice per group. **P* < .05, ***P* < .01 vs IgG control. Abbreviations: b.d.= below detection, n.d= not determined.

| 1 | Gender | Age |  | Occupation |
| --- | --- | --- | --- | --- |
| 2 | Diabetes mellitus | Liver cirrhosis | Malignancy | Malnutrition* |
| 4 | Melioidosis | Thrombocytopenia |  |  |
| 5 | Bleeding | Immunothrombosis | Sepsis |  |
|  | Respiratory failure | Hypotension | Kidney failure |  |
| 6 |  | Mortality |  |  |

**S1 Fig. Conceptual hierarchical framework for risk factors for thrombocytopenia and mortality.**

Gender, age and occupation occupy the highest level in the hierarchy because they are not dependent on any other factors. Factors in each level are dependent on factors in the level above and factors in lower levels cannot confound the effect of factors in higher levels because they occur later in time; a conceptual hierarchical framework its built around social and biological plausibility of which came first in time [1]. Factors in level 4 are immediate proximate causes of death. *We used rice farming (occupation) as a proxy for malnutrition.

**S2 Fig. *B. pseudomallei* growth in blood during experimental melioidosis.** Mice were treated with anti-GPIbα or IgG control (0.4µg/g), infected with *B. pseudomallei* via the airway and sacrificed after 24, 48 or 72 hours. Bacterial quantification in blood. Data are expressed as box-and whisker plots depicting the smallest observation, lower quartile, median, upper quartile and largest observation. N=8 mice per group.

**A B**

**S3 Fig. *Ex vivo* *B. pseudomallei* growth.** (A) Mice were treated with anti-GPIbα (0.4µg/g, platelet depletion) or IgG control (0.4µg/g) and sacrificed uninfected. Whole blood was incubated with viable *B. pseudomallei* (10^7^CFU/mL) for 20 hours at 37 degrees after which bacterial counts were quantified. (B) Human platelet poor and platelet rich plasma was incubated with viable *B. pseudomallei* (5*10^6^CFU/mL) for 20 hours at 37 degrees after which bacterial growth was quantified. Data are expressed as box-and whisker plots depicting the smallest observation, lower quartile, median, upper quartile and largest observation. N=4 replicates or mice per group. Human experiments were performed at least twice with 2 independent donors.

**S4 Fig. Platelet depletion does not influence total cell influx in the bronchoalveolar space during experimental melioidosis.** Mice were treated with anti-GPIbα (platelet depletion) or IgG control (0.4µg/g), infected with *B. pseudomallei* via the airway and sacrifice after 72 hours or uninfected. Total cell numbers in the BALF. Data are expressed as box-and whisker plots depicting the smallest observation, lower quartile, median, upper quartile and largest observation. N=8 mice per group. Abbreviation: BALF= bronchoalveolar lavage fluid.

**
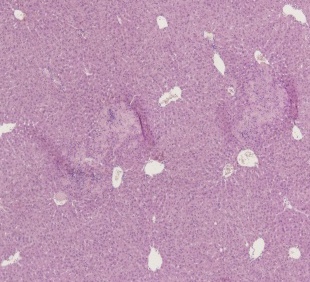

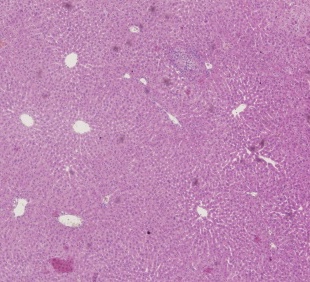

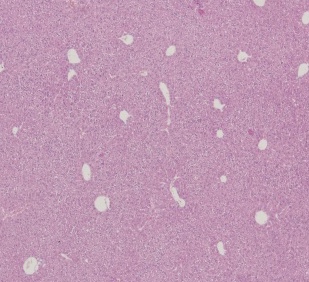
**

**A**

**B**

**C**

**D**

t=72 BPS, Control

Uninfected, Platelet depletion

t=72 BPS, Platelet depletion

**S5 Fig. Platelet depletion does not influence liver damage.** (A-D) Mice were treated with anti-GPIbα (platelet depletion) or IgG control (0.4µg/g), infected with *B. pseudomallei* via the airway and sacrificed after 24, 48 or 72 hours. Representative images, original magnification 40x (A) and quantification (B) of liver damage. ALT (C) AST (D) plasma levels. Data are expressed as box-and whisker plots depicting the smallest observation, lower quartile, median, upper quartile and largest observation. N=8 mice per group. **P* < .05 vs IgG control. Abbreviations: BPS= *Burkholderia pseudomallei*, ALT= alanine transaminase and AST= aspartate transaminase.

**A**

**B**

**C**

**S6 Fig. Platelet depletion <1% also impairs host defense and vascular integrity during melioidosis.**  (A-C) Mice were treated with high dose anti-GPIbα 2µg/g or IgG control and infected with *B. pseudomallei* via the airway and sacrificed after 24, 48 or 72 hours. Bacterial loads in organs indicated (A). Blood platelet counts, MPO levels lung, Ly6G staining lung, and liver pathology (B). ALT, AST levels, lung bleedings score, and lung hemoglobin levels (C). Data are expressed as box-and whisker plots depicting the smallest observation, lower quartile, median, upper quartile and largest observation. N=8 mice per group. **P* < .05, ***P* < .01, *** *P* <.001 vs IgG control. Abbreviations: ALT= alanine transaminase and AST= aspartate transaminase.

T=24 Control BPS

T=24 Platelet depletion BPS

Uninfected Control

T=72 control infected


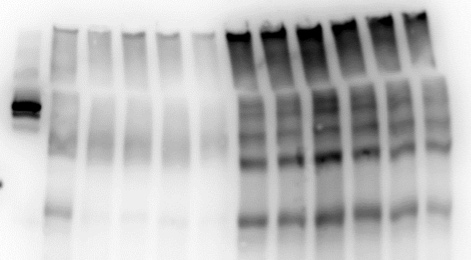


NP +

- Fibr multimer

- Fibrinogen

- Fragment X

- D-dimer


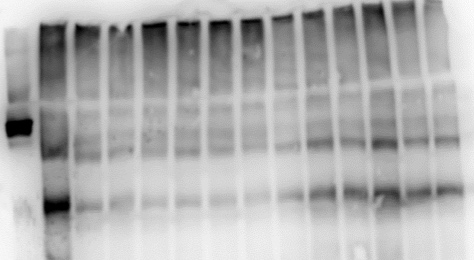


- Fibr multimer

- Fibrinogen

- Fragment X

- D-dimer

NP +


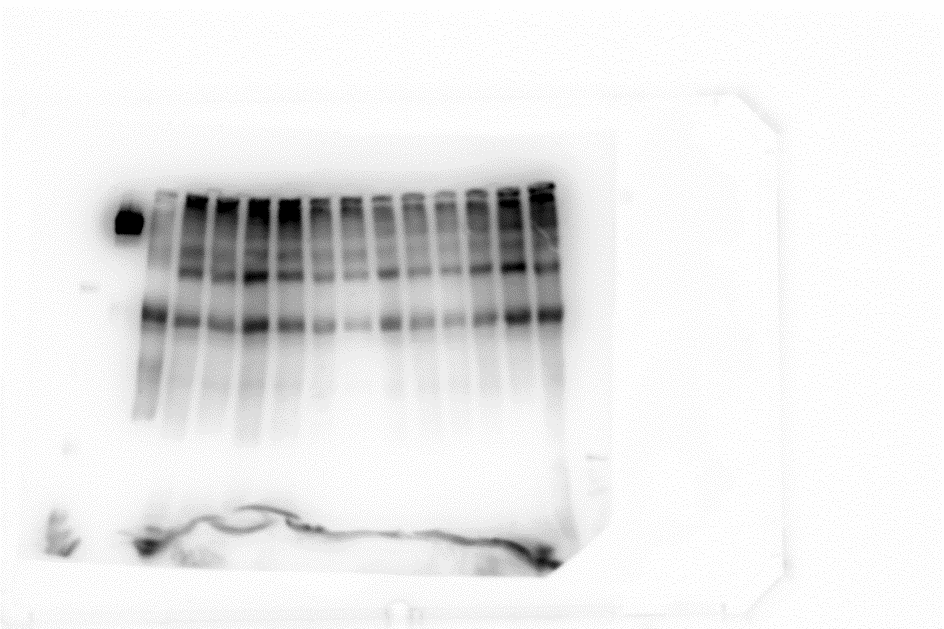


T=72 Control BPS

T=72 Platelet depletion BPS

- Fibr multimer

- Fibrinogen

- Fragment X

- D-dimer

NP +

**B**

**D**

**C**

**A**

**S7 Fig. Thrombocytopenia results in increased local and systemic coagulation.** (A-D) Mice were treated with anti-GPIbα (platelet depletion) or IgG control (both 0.4µg/g) and infected with *B. pseudomallei* via the airway and sacrificed after 24, 48 or 72 hours or sacrificed uninfected. Plasma and lung thrombin-anti-thrombin complex (TATc) levels (A). Lung fibrinogen western blot showing D-dimer, and semi quantification D-dimer in uninfected mice (B). Lung fibrinogen western blots and semi quantification of D-dimer in mice infected for 24 hours (C) and 72 hours (D). Data are expressed as box-and whisker plots depicting the smallest observation, lower quartile, median, upper quartile and largest observation or as bars (mean and SEM). N=8 mice per group. **P* < .05 and ***P* < .01 vs IgG control. Abbreviations: NP= naïve plasma, +=positive D-dimer control.

**A**

**B**

**S8 Fig. Platelet Toll-like receptor (TLR) signaling does not influence host defense during murine melioidosis.**  (A-B) Plt-Myd88^-/-^ (white boxes) or control mice (grey boxes) were infected with *B. pseudomallei* via the airway and sacrificed after 72 hours. Bacterial quantification in indicated organs (A). Platelet counts in blood, platelet P-selectin expression, and platelet-neutrophil complex formation in blood (B). Data are expressed as box-and whisker plots depicting the smallest observation, lower quartile, median, upper quartile and largest observation. N=8 mice per group.

**** **A B**

**D**

**C**

**E**

**
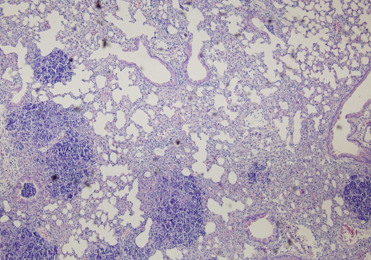

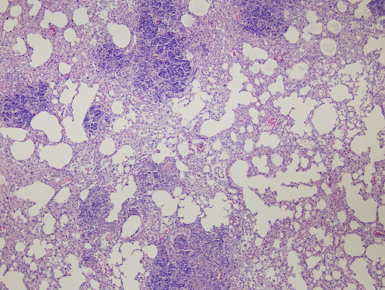
**

t=72 BPS, Control

t=72 BPS, IIL4R/GPIbα

**S9 Fig: Lung bleeding in IL4R/GPIbα, Plt-Myd88^-/-^ and control mice.** (A) IL4R/GPIbα or control mice were infected with *B. pseudomallei* via the airway and sacrificed after 72 hours and hemoglobin levels were determined in lung homogenates. (B) Plt-Myd88^-/-^ or control mice were infected with *B. pseudomallei* via the airway and sacrificed after 72 hours and hemoglobin levels were determined in lung homogenates. Lung pathology quantification (C), lung bleeding score (D) and representative images original magnification 40x of IL4R/GPIbα and control mice. Data are expressed as box-and whisker plots depicting the smallest observation, lower quartile, median, upper quartile and largest observation. N=8 mice per group. Abbreviations: OD= optical density and BPS= *Burkholderia pseudomallei.*

**References**

1. Victora CG, Huttly SR, Fuchs SC, Olinto MT. The role of conceptual frameworks in epidemiological analysis: a hierarchical approach. International journal of epidemiology **1997**; 26:224-7.
