# Supplementary material for: Thrombocytopenia Impairs Host Defense Against Burkholderia pseudomallei (Melioidosis)
Source: J Infect Dis. 2018 Oct 11;219(4):648–59. doi: 10.1093/infdis/jiy541 (PMC6350952; doi:10.1093/infdis/jiy541)
Supplement: Supplementary Material and Methods [file jiy541_supplemental_material_methods.docx]

**Supplementary Material**

**Methods**

*Cohort study*

Patients were eligible if it was their first admission for culture-confirmed melioidosis, with patients younger than 15 years of age excluded (because pediatric cases have a different clinical presentation and prognosis). There were no other exclusion criteria. Outcome in the group with admission platelet counts <50x10^9^/L and 50–100x10^9^/L were not different (the confidence interval for one group was entirely contained within the confidence interval for the other). Analysis of other parameters did not reveal relevant differences between these groups. The results reported are therefore for the two groups combined for all analyses. The primary study outcome was in-hospital mortality, but we also pre-defined three secondary outcomes: hypotension (a systolic blood pressure of less than 90 mmHg at any point during admission), acute kidney injury (diagnosed by attending physicians) and respiratory failure (hypoxia judged clinically to require mechanical ventilation; arterial blood gases are not taken routinely in our setting).

*Animals*

Human IL4R/GPIbα mice (University of Arkansas for Medical Sciences, Little Rock, USA) are knock out for mouse GPIbα, without the associated macrothrombocytopenia that is prevented by transgenic expression of a protein chimera that consists of the extracellular part of the IL-4 receptor and the intracellular part of GPIbα [1]. Control mice were kept with co-housing. All genetically modified mice were backcrossed > 6 times to a C57Bl/6 genetic background. Mice were housed in Animal Research Institute AMC facility under standard care and received standard rodent chow and water ad libitum. All experiments were conducted with mice between 8 and 12 weeks of age. Samples were randomized if applicable.

*Experimental study design*

The clinical observation score consisted of the following parameters: solitude (0, absent; 1, present), posture (0, normal; 1, sphere), fur (0, normal; 1, pilo-erection), eyes (0, open; 1, closed; 2, dirty), alertness (0, normal; 1, slow; 2, apathetic; 3, non-responsive), pace (0, normal; 1, shaky; 2, collapse), respiration (0, normal; 1, heavy; 2, slow; 3, intermittent) and time to ascent when laid down (0, normal; 1, < 5 s; 2, > 5 s; 3, unresponsive). Mice were euthanized 24, 48 or 72 hours after induction of infection (n=8 per group); non-infected mice were sacrificed simultaneously (n=4 per group). Lungs for pathology and bronchoalveolair lavage fluid (BALF) were obtained in separate experiments to avoid dilution of samples.

*Flow cytometry*

Murine whole blood samples were assessed by flow cytometry (FACS Calibur, Becton Dickinson, Franklin Lakes, NJ, USA). Murine platelet counts were measured using hamster anti-mouse-CD61 mAb (BioLegend, San Diego, CA). GpIb expression was assessed by anti-mouse GPIba (Clone:Xia3, Emfret analystics). Platelet–neutrophil complex formation was determined by using rat anti-mouse-CD11b mAb (BD Biosciences, San Diego, CA, USA), rat anti-mouse-CD115 mAb (eBioscience, San Diego, CA, USA) and rat-anti-mouse Ly-6G&C mAb (BD Biosciences) in combination with anti-CD61 mAb.

*Pathology*

The paraffin embedded left lung lobe was cut into four-micrometer sections and stained with hematoxylin and eosin (H&E). Slides were coded and scored by a pathologist blinded for group identity as previously described [2,3]. In the lung bleeding was scored (0-4) and in the liver the following parameters were scored 0-4: inflammation, presence of necrosis/abscess formation, presence of thrombi and bleeding. The total histopathology score was calculated as the sum of the scores of all individual parameters. After staining, expression was quantified by digital image analysis: slides were scanned with the Olympus Slide system (Olympus dotSlide, Tokyo, Japan) to generate TIFF images of the full tissue section. To determine neutrophil influx in the lung, sections were stained with anti-mouse Ly6-G mAb (BioLegend). Ly-6G positivity was measured using Image J (U.S. National Institutes of Health, Bethesda, MD); the amount of positivity was expressed as percentage of the total lung surface area.

*Protein measurements*

Interleukin (IL)-6, TNF-α, CCL2 and interferon (IFN)-y were determined with a commercially available cytometric beads array multiplex assay (BD Biosciences). Myeloperoxidase (MPO) and CXCL2 were measured by ELISA (all R&D systems, Minneapolis, MN) as well as TATc levels (Affinity Biologicals Inc., Hamilton, Canada). Fibrin products were determined by western blot by using rabbit anti-mouse fibrinogen antibody (MyBioSource.com, San Diego, CA). Positive control for D-dimer was generated as previously described [4]. Hemoglobin concentrations were measured in 50-fold diluted lung homogenates by light density at 410 nm by NanoDrop spectrophotometer (Thermo Fisher Scientific). AST and ALT were measured using a c702 Roche Diagnostics (Roche Diagnostics BV, Almere, the Netherlands). cfDNA was determined by diluting samples 50-100 fold with PBS containing 0.1% BSA and mixed with an equal volume of 1 μM SytoxGreen (Thermo Scientific, Waltham, MA, USA). CitH3 levels were determined by western blot using rabbit-anti-citH3 (Abcam, Cambridge, UK). Immunoreactive bands were visualized using an ImageQuant LAS 4000 (FujiFilm™ Corporation, Tokyo, Japan). For quantification, densitometry was performed with Adobe Photoshop (Adobe Systems Incorporated, San Jose, CA).

*Statistics of murine experiments*

For murine studies, data are expressed as box and whisker plots or as bars (respectively median with range or mean with SD). Comparisons between groups were first performed using a one-way analysis of variance on ranks (ANOVA); only when significant differences were present, groups at individual time points were tested using the Mann-Whitney U test. Survival was compared using the Kaplan-Meier method, followed by the log-rank test. Clinical observation scores were compared with a repeated measure analysis of variance. Analyses were done using GraphPad Prism version 7.0 (GraphPad Software, San Diego, CA). P-values < 0.05 were considered statistically significant.

**References**

1. Ware J, Russell S, Ruggeri ZM. Generation and rescue of a murine model of platelet dysfunction: the

Bernard-Soulier syndrome. Proc Natl Acad Sci U S A 2000; 97:2803-8.

2. de Stoppelaar SF, van 't Veer C, Claushuis TA, Albersen BJ, Roelofs JJ, van der Poll T. Thrombocytopenia

impairs host defense in gram-negative pneumonia derived sepsis in mice. Blood 2014; 124:3781-90.

3. Wiersinga WJ, de Vos AF, de Beer R, et al. Inflammation patterns indcued by different Burkholderia

species in mice. Cell microbiol 2008; 10:81-7.

4. Claushuis TA, de Stoppelaar SF, Stroo I, et al. Thrombin contributes to protective immunity in

pneumonia-derived sepsis via fibrin polymerization and platelet-neutrophil interactions. Journal of

thrombosis and haemostasis: JTH 2017; 15:744-57.
